# A Phylogeographic Analysis of Porcine Parvovirus 1 in Africa

**DOI:** 10.3390/v15010207

**Published:** 2023-01-11

**Authors:** Giovanni Franzo, Habibata Lamouni Zerbo, Bruno Lalidia Ouoba, Adama Drabo Dji-Tombo, Marietou Guitti Kindo, Rasablaga Sawadogo, Jelly Chang’a, Stella Bitanyi, Aloyce Kamigwe, Charles Mayenga, Modou Moustapha Lo, Mbengué Ndiaye, Aminata Ba, Gaye Laye Diop, Iolanda Vieira Anahory, Lourenço P. Mapaco, Sara J. Achá, Valere Kouame Kouakou, Emmanuel Couacy-Hymann, Stephen G. Gacheru, Jacqueline K. Lichoti, Justus K. Kasivalu, Obadiah N. Njagi, Tirumala B. K. Settypalli, Giovanni Cattoli, Charles E. Lamien, Umberto Molini, William G. Dundon

**Affiliations:** 1 Department of Animal Medicine, Production and Health, University of Padova, viale dell’Università 16, 35020 Legnaro, Italy; 2 Laboratoire National d’Elevage (LNE), Ouagadougou 03 BP 907, Burkina Faso; 3 Centre for Infectious Diseases and Biotechnology, Tanzania Veterinary Laboratory Agency, Dar es Salaam P.O. Box 9254, Tanzania; 4 Laboratoire National de l’Elevage et de Recherches Vétérinaires, Institut Sénégalais de Recherches Agricoles (ISRA), Dakar BP 3120, Senegal; 5 Central Veterinary Laboratory, Agricultural Research Institute of Mozambique, Directorate of Animal Science, Maputo 1922, Mozambique; 6 Centre National de Recherche Agronomique (CNRA), Abidjan 1740, Côte d’Ivoire; 7 Central Veterinary Laboratory, Directorate of Veterinary Services, Kabete P.O. Box 00100-34188, Kenya; 8 Animal Production and Health Laboratory, Animal Production and Health Section, Joint FAO/IAEA Division, Department of Nuclear Sciences and Applications, International Atomic Energy Agency, P.O. Box 100, 1400 Vienna, Austria; 9 School of Veterinary Medicine, Faculty of Health Sciences and Veterinary Medicine, University of Namibia, Neudamm Campus, Windhoek Private Bag 13301, Namibia; 10 Central Veterinary Laboratory (CVL), 24 Goethe Street, Windhoek Private Bag 18137, Namibia

**Keywords:** porcine parvovirus 1, Africa, epidemiology, phylogeography, phylogeny, VP2

## Abstract

Porcine parvovirus 1 (PPV1) is recognized as a major cause of reproductive failure in pigs, leading to several clinical outcomes globally known as SMEDI. Despite being known since the late 1960s its circulation is still of relevance to swine producers. Additionally, the emergence of variants such as the virulent 27a strain, for which lower protection induced by vaccines has been demonstrated, is of increasing concern. Even though constant monitoring of PPV1 using molecular epidemiological approaches is of pivotal importance, viral sequence data are scarce especially in low-income countries. To fill this gap, a collection of 71 partial VP2 sequences originating from eight African countries (Burkina Faso, Côte d’Ivoire, Kenya, Mozambique, Namibia, Nigeria, Senegal, and Tanzania) during the period 2011–2021 were analyzed within the context of global PPV1 variability. The observed pattern largely reflected what has been observed in high-income regions, i.e., 27a-like strains were more frequently detected than less virulent NADL-8-like strains. A phylogeographic analysis supported this observation, highlighting that the African scenario has been largely shaped by multiple PPV1 importation events from other continents, especially Europe and Asia. The existence of such an international movement coupled with the circulation of potential vaccine-escape variants requires the careful evaluation of the control strategies to prevent new strain introduction and persistence.

## 1. Introduction

Porcine parvovirus 1 (PPV1) is a virus classified in the species *Ungulate protoparvovirus 1* of the genus *Protoparvovirus* in the virus family *Parvoviridae* (https://ictv.global/taxonomy, accessed 10 January 2023). It is a non-enveloped virus with a single-stranded DNA genome of about 5 kb including two main coding regions that encode non-structural (NS1, NS2, and NS3) and structural (VP1, VP2, and VP3) proteins [1,2]. PPV1 has been recognized as the etiological agent of reproductive disorders in swine for a long time, globally known as SMEDI (i.e., stillbirth, mummification, embryonic death, and infertility), causing significant economic losses worldwide [1,2,3]. Similar to other ssDNA viruses, PPV1 is characterized by a relatively high evolutionary rate, ranging from 10^−6^ to 10^−4^ substitutions∙site^−1^∙year^−1^, depending on the cited study and the genomic region [1,4,5]. This rapid evolution has generated significant genotypic and phenotypic variability over time that has been classified according to different systems proposed by different authors [6,7,8] (sub-species classification has not yet been well standardized).

Such heterogenicity has been associated with variation in tropism and virulence, whose determinants are most likely confined to the structural proteins. For example, among the VP2 amino acids, three of them, and their relative heterogeneity (i.e., D378G, H383Q, and S436P), have been considered to be responsible for different tissue tropism [9,10]. Similarly, viral variability could negatively impact neutralizing antibodies’ pivotal role in host protection. Cross-protection among strains has been assessed in experimental studies, revealing higher protection against homologous compared to heterologous challenge [11]. The lower affinity to neutralizing antibodies has been linked to amino acid substitution in the 3-fold spike region. 

Vaccines against PPV1 have been used since the early 80s and have largely been administered in the last 30 years [5]. However, despite broad application, an increase in SMEDI cases has been reported in the last few years in Europe [5]. This increase is often associated with the new variant 27a or 27a-like strains that have become predominant in Europe [5,6]. It has been suggested that the appearance of 27a over the last decades is the result of viral adaptation to vaccine pressure on a viral population circulating in a partially immune population [4,5,8]. Overall, a reduction in viral diversity in favor of viruses more able to deal with vaccine-induced pressure has been postulated. In silico and in vitro analyses, plus the evidence that strains detected in wild boars showed a higher variability compared to their domestic counterparts, seem to confirm such a hypothesis [8].

Although the consequences in terms of clinical protection are far more debated [12], the implications that potential differential cross-protection has on PPV1 epidemiology justifies a constant updating of the molecular epidemiology of the virus. Nevertheless, this information is still limited and biased according to temporal and spatial distribution. Data from Africa are especially scarce even though such knowledge is of particular relevance for several reasons: the impact of the productive losses on society with already limited resources; the growing economic and commercial relationship of many African countries with more developed regions; the characteristics of the African farming system, its heterogeneity and the frequent contact opportunities with wild species, which could enhance the persistence, circulation, and evolution of strains with unusual features, as demonstrated for other swine pathogens [13]. Unfortunately, limited resources and capacity often prevent significant diagnostic and sequencing efforts in many African countries.

The present study aimed to provide, despite these limitations, an as extensive as possible characterization of PPV1 strains in Africa and evaluate the potential introduction sources thereby providing important data for local and regional veterinary authorities involved in porcine disease management.

## 2. Materials and Methods

### 2.1. Swine Samples

Archived DNA purified from samples (i.e., spleen, lung, liver, blood, serum) collected from pigs as part of routine diagnostic activities in Burkina Faso (*n* = 52), Ivory Coast (*n* = 54), Kenya (*n* = 9), Mozambique (*n* = 96), Senegal (*n* = 17), and Tanzania (n = 123) between 2011 and 2021 (Table S1) were screened by PCR for the presence of PPV1 as previously described [14]. Positive amplicons of a 739 bp region of the VP2 were purified and sequenced commercially by LGC Genomics (Berlin, Germany)

### 2.2. Sequence Analysis

PPV1 nucleotide sequences spanning the same VP2 region obtained in the present study and originating from Europe, North and South America, and Asia were downloaded from GenBank (when the sampling country and date were available). In addition, sequences (*n* = 40) from two African countries Namibia and Nigeria were included [14,15,16]. All the sequences were merged with the ones generated in the present study and aligned using MAFFT [17] and their quality was evaluated. Partial or poorly aligned sequences, those displaying unknown bases, premature stop codons, or frameshift mutations were excluded from further analysis. Recombination analysis was performed using GARD [18] and RDP4 [19] to identify and remove recombinant strains from the dataset. RDP4 analysis settings were selected based on the dataset features according to the recommendations of the RDP manual. A recombination event was accepted as significant if detected by more than two methods with a significance level of 0.05 after Bonferroni correction. The presence of adequate phylogenetic and temporal signals was tested using the likelihood mapping approach implemented in IQ-Tree [20] and the TempEst [21] programs, respectively. A phylogenetic tree was reconstructed using IQ-Tree selecting the substitution model with the lowest Akaike information criterion (AIC) score calculated using the same software and assessing the robustness of detected clades performing 1000 bootstrap replicates.

### 2.3. Viral Population Dynamics and Phylogeography

Considering that the sequence selection was biased for collection country and date, more balanced datasets were obtained by randomly subsampling a maximum of three sequences per country-year. To assess the effect of sampling, five random datasets were generated and independently analyzed. PPV1 population parameters, including time to the most recent common ancestor (tMRCA), evolutionary rate, and population size variation over time were estimated using the Bayesian serial coalescent approach implemented in BEAST 1.10.4 [22]. The nucleotide substitution model was selected based on the Bayesian information criterion (BIC) calculated using JModelTest2 [23] while the best-fitting molecular clock model was selected by calculating the Bayesian factor (BF) estimating the marginal likelihood of the evaluated models using the path sampling (PS) and stepping stones (SS) methods [24]. The non-parametric Skygrid [25] model was selected to reconstruct the trend of the relative genetic diversity (i.e., effective population size × generation time; Ne × t) over time. Strain migration among countries was estimated using the discrete state phylogeographic approach [26]. The Bayesian stochastic search variable selection (BSSVS) was also implemented to allow for the identification of the most parsimonious description of the phylogeographic diffusion process and to construct a BF test assessing the statistical significance of such links. All parameters were jointly estimated using a 100 million generation Markov chain Monte Carlo (MCMC) chain, sampling the population parameters and trees every 10 thousand generations. Run performances were summarized and evaluated using Tracer 1.7 after removing the first 20% as burn-in. Run results were accepted if the estimated sample size (ESS) was higher than 200 and the mixing and convergence, evaluated by visual inspection of the run’s trace, were adequate. A maximum clade credibility tree (MCC) was obtained using the Treeannotator suite of the BEAST package. SPREAD3 [27] was used to identify the statistically supported migration rates between country pairs. The significance level was set to BF > 10 for all considered analyses. Additional summary statistics and graphics were generated using R [28] and specific libraries [29,30].

## 3. Results

Of the 351 samples screened by PCR in this study, 31 (8.8%) were positive for PPV1 [i.e., Burkina Faso (*n* = 2; positivity ratio = 3.85%), Ivory Coast (*n* = 7; positivity ratio = 12.96%), Kenya (*n* = 1; positivity ratio = 11.11%), Mozambique (*n* = 17; positivity ratio = 17.71%), Senegal (*n* = 2; positivity ratio = 11.76%), Tanzania (*n* = 2; positivity ratio = 1.63%)]. Additionally, 40 sequences previously obtained from Namibia (positivity ratio = 36.35%) and Nigeria (positivity ratio = 20.6%) were included in the study [14,15,16].

In total, 71 sequences originating from eight African countries (Burkina Faso, Ivory Coast, Kenya, Mozambique, Namibia, Nigeria, Senegal, Tanzania) in the period 2011–2021 were analyzed in the present study (Table S1). The obtained sequences covered a region from position 3035 to 3608 of the U44978 reference genome. After merging all of the African sequences with the other sequences available in GenBank, 312 sequences from 24 countries sampled between 1963 and 2021 were included in the final dataset (Table S2). Phylogenetic and temporal signals were adequate for further analysis. The average distance among the strains was 1.00% (interval: 0–4.2%) while it ranged between 0 and 3% (average = 0.7%) when considering the African strains only (Table S3). No significant recombination event was detected in the region considered using the selected analysis settings.

Overall, 47 African strains were related to the 27a strain (Cluster PPV1b, according to the Vereecke et al., classification), 21 to the NADL-8 (Cluster PPV1d), and three to the Cluster PPV1a (Figure S1).

**Figure 1:**
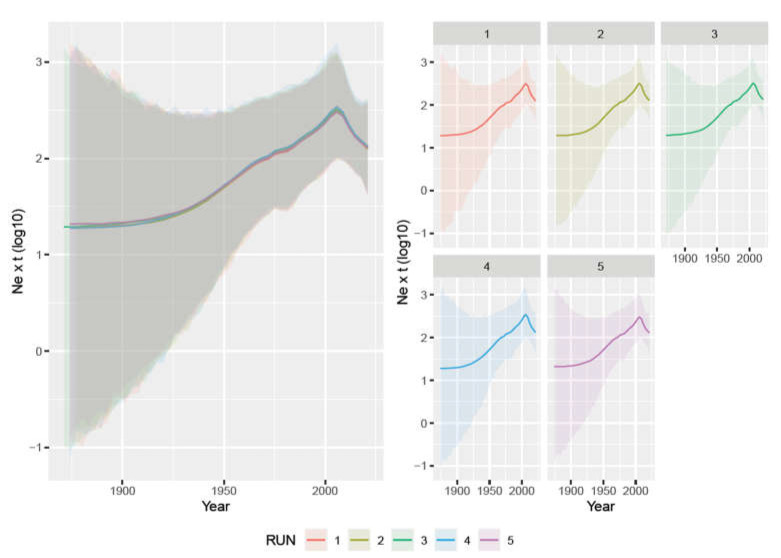
Left figure: mean relative genetic diversity (Ne × t) of the worldwide PPV1 population over time. The results of the five independent runs have been color-coded. Right figure: mean and upper and lower 95HPD values are reported for each run.

The analysis of the five independent datasets provided highly concordant results. The tMRCA was estimated in 1918.08 [95HPD: 1872.06–1952.65] (average of the five datasets) and the evolutionary rate was 1.735 × 10^−4^ [95HPD: 7.944 × 10^−5^–3.12 × 10^−4^]. The viral population size demonstrated a constant increase from the tMRCA until approximately 2010 when a progressive decrease was observed (Figure 1).

The phylogeographic analysis highlighted several well-supported migration rates connecting African countries with others, especially from Asia and Europe (Figure S2). More specifically, although with minor differences among datasets, significant connections linked Denmark with Mozambique, Ivory Coast, and Tanzania. Mozambique had also significant connections with China and other African counties such as Namibia and Tanzania. Finally, a connection between Senegal and South Korea and the USA was detected. Other links involving African counties were also present, although they did not reach the fixed significance level. Phylogenetic tree analysis highlighted a close relationship between Namibian and Mozambican strains and between strains from Nigeria, Ivory Coast, and Mozambique (Figure S1). More specifically, based on the ML phylogenetic tree, it was possible to identify different clades to which African strains belonged (herein named Clade A-H; see Figure S1). Clade A included two strains from Ivory Coast and one from Tanzania, in addition to European (mostly from Denmark but also Germany, Ireland, and Romania) and Asian (China) strains. Clade B, including one strain from Tanzania, was composed essentially of strains from Asia, i.e., China and South Korea). Clade C comprised strains from Namibia and Mozambique plus three strains from Romania. Clade D included Nigerian strains only, although a certain relationship with European strains was observed. Clade E included strains from Mozambique, Denmark, and the Netherlands. Clade F included Namibian and Denmark strains only. Clade G included European sequences plus one from Burkina Faso. Finally, Clade H, although genetically homogenous, was highly heterogenous in terms of the countries from which the strains were collected, since it included viruses from Ivory Coast, Kenya, Nigeria, and Mozambique, in addition to European (i.e., Denmark, Romania, the Netherlands, France, Belgium, Germany, and Spain) and Asian (i.e., China, India, and South Korea) countries. One strain from Mozambique, Ivory Coast, Kenia, Burkina Faso, and two from Senegal were not part of a well-defined clade, although they were closely related to Asian and European strains.

Therefore, several countries harbored strains belonging to different clades, highly suggestive of multiple introduction events: Burkina Faso (2 clades), Ivory Coast (4 clades), Mozambique (3 clades), Namibia (2 clades), Nigeria (2 clades), and Tanzania (2 clades).

**Figure 2:**
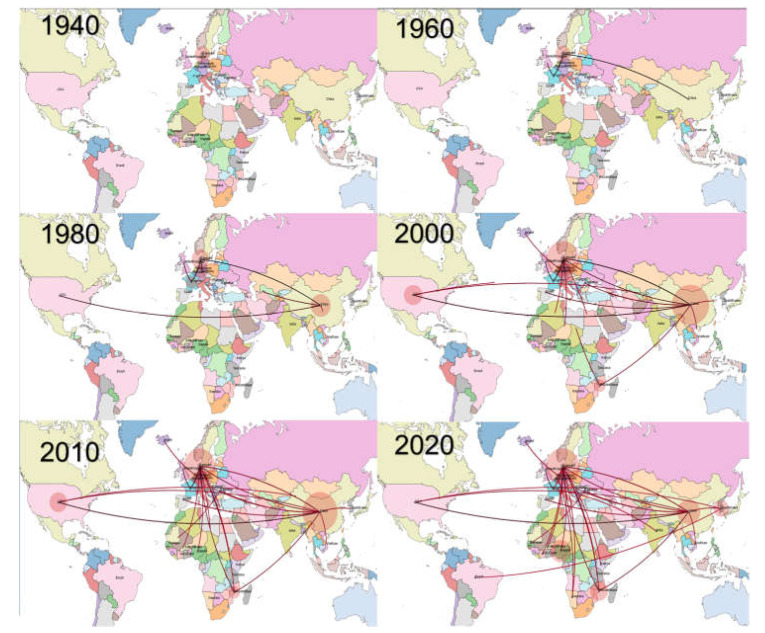
Phylogeographic reconstruction of PPV1 migration among countries over time. Each picture represents a different decade. The edges connecting the countries have been color-coded from black to red based on the estimated age.

Viral dispersal over time suggested a probable European origin of the virus (Figure 2 and Figure S3), where it persisted until the 1960s and thereafter migrated to Asia and North America in the following twenty years. Since the beginning of the new millennium, Asia and Europe emerged as the main sources of viral dispersal and introduction into African countries. Within Africa spreading was also observed, although with higher uncertainty and variability among datasets.

## 4. Discussion

This study represents the first attempt to investigate PPV1 molecular epidemiology in Africa and contextualize it within a worldwide scenario. The obtained results describing the evolutionary dynamics of PPV1 are in complete agreement with those of previous studies. Similarly, the high repeatability of output data using the independently generated datasets testifies that sampling bias does not significantly affect the present inferences, supporting the reliability of our results. The estimated substitution rate was approximately 10^−5^ substitutions∙site^−1^∙year^−1^, which reflects other authors’ work [4,5] and highlights the high evolutionary potential of PPV1, as observed for several other ssDNA viruses. As reported by Vereecke et al., using VP2 sequences, PPV1(tMRCA) was predicted at the beginning of the previous century and, similarly to what transpired for other pig pathogens, a constant increase in the population size occurred [5]. This highlights a prolonged, undetected viral circulation in the European swine population which initially likely caused limited damage. The progressive intensification of farming systems sustained the increase in viral circulation and prevalence and thereafter its global spread [31,32]. Modern farming conditions likely contributed, together with the emergence of other co-infections and predisposing factors, to the emergence of PPV1 as an economically relevant, clinically overt disease. A successive decrease in the viral population size was predicted from the mid-2010s, in agreement with Vereecke et al. [5]. Such evidence is of particular interest since the first vaccines were introduced in the early 1980s, although adequate vaccination coverage was reached only years later and with high variability among countries. The long latent period between vaccine introduction and its effect on viral circulation, although also most probably confounded by the parallel increase in swine populations that sustained an increase in the viral population, testify to the need for extensive immunization campaigns and high population coverage to achieve successful results. Additional improvements in biosecurity measures and the better control of other co-infections (e.g., PCV-2, PRRSV) that occurred in those years probably also had a direct impact on PPV1 dynamics.

Viral circulation in a partially immune environment has also been proposed to be involved in the evolution of PPV1 and the emergence of vaccine escape variants [5]. Particular attention has been paid to the 27a strain that has become predominant in Europe and for which lower protection from infection with currently available vaccines has been demonstrated [1]. Most of the African strains were closely related to this variant and, to a lesser extent, to NADL-8. The phylogeographic analysis confirms these findings since several links were estimated between African countries and Europe or Asia. Therefore, the evolution of the epidemiological scenario in these regions directly affected the African one. Overall, several clades that included strains collected in Africa were identified. Some of these clades consisted of strains identified in Asian and European countries, strengthening the evidence for intense worldwide circulation of PPV1 and the role of these regions in strain importation in Africa. Of note, strains collected in the African countries were often part of different, poorly related clades, which is highly suggestive of multiple introduction events. Denmark, and to a lesser extent China, emerged as the most common origins of viral dispersal. However, the limitations in data availability and the close genetic relationship among strains circulating in Europe and Asia make it difficult to identify specific links and to establish specific sources of virus since other countries seemed to be involved both directly or as part of more extensive, undetected, transmission chains. Therefore, it is often challenging to understand if the clustering of African strains with specific countries is due to real epidemiological links or sequence paucity. Caution should therefore be exercised when interpreting connections between countries in this context. Nevertheless, the overall pattern can be assumed with a certain degree of confidence, being also supported by epidemiological evidence. Live swine or semen importation in Africa, although not common, occurred (https://www.fao.org/faostat/) especially in periods when internal sources such as South Africa were excluded from trade due to African Swine Fever (2005 and 2016) and Foot and Mouth disease outbreaks (2019 and 2022). Moreover, significant numbers of pork products were also imported by South Africa which, in turn, was the main source of exportation to other African countries [33]. Unfortunately, the lack of data from this region prevents definitive conclusions and so, more intensive sampling and sequencing are recommended. Interestingly, several introduction events were predicted in the early 2000s, which is compatible with the above-mentioned hypothesis. Similarly, Asia and China in particular, have played an increasing role in African countries’ economies [34], including the agricultural sector, thus increasing the risk of direct or indirect contact between animals and their by-products. Similar connections have been reported for several infections affecting both companion animals and livestock [35,36]. The high environmental resistance of PPV1 could also point to more obscure, indirect, and long-distance transmission pathways. Moreover, pig by-products are still commonly used for animal nutrition in several African farms and could thus represent an important source of virus importation from foreign countries. Once introduced, some African strains were detected more than once, testifying to the establishment of successful and persistent infections. Although some links between African countries were also present, they were rare and involved mostly neighboring countries, which suggests that PPV1 epidemiology in Africa is mainly shaped by strain introduction (even through multiple events) from non-African countries followed by local evolution, rather than intra-continental spread. However, the low number of African countries for which sequences were available for this study could conceal a more complex scenario.

The present study has several limitations. The first is ascribable to the overall scarcity and biased nature of global PPV1 molecular epidemiology data. The good concordance among the randomly generated datasets and with other studies allows for confidence in the obtained results, at least when interpreted in terms of overall trends and patterns. On the other hand, we discourage any overstatement of fine-level interaction and connection between country pairs since other links could be concealed by undersampling.

The other main limitation is that only partial VP2 sequences were obtained from a limited number of African countries, which prevented an in-depth investigation of phenotypic features and association with viral clinical/biological features. While we recognize that complete VP2 sequences from a higher number of regions would have been preferable to allow a better characterization of African PPV1 molecular epidemiology and evaluate the potential determinants of virulence, tropism, and cross-protection [9], we also need to stress the challenges of undertaking a standardized research project involving several countries with different priorities and limited resources. Therefore, this study was mainly based on the samples collected during non-specific animal disease diagnostic activities. We hope that these results, although preliminary and improvable, will prompt new efforts to refine such investigations.

Despite these shortcomings, it was possible to imply the circulation of several PPV1 strains characterized by significant genetic variability, largely originating from multiple introductions from Europe and Asia. Most of the strains were closely related to virulent strains, including the 27a, for which a sub-optimal cross-protection conferred by the currently available vaccine has been proven. Currently, PPV1 vaccination is extremely rare in Africa, even in intensive farms due to economical constraints. Although current vaccines still appear to be beneficial in the control of clinical disease, their introduction should be carefully considered in light of their limitations and the peculiarities of the African scenario. Therefore, significant efforts should be made to decrease the risk of the introduction of new strains, evaluate the efficacy of vaccination, assess the economic impact of PPV1 in the African context, and estimate the cost–benefits of different control strategies to allow a proper prioritization of limited resources.

## Data Availability

Sequence accession numbers and datasets are reported in Supplementary material.

## Supplementary Materials

The following supporting information can be downloaded at: https://www.mdpi.com/article/10.3390/v15010207/s1, Figure S1: Maximum likelihood phylogenetic tree based on the partial VP2 gene. The collection continents have been color-coded. Figure S2: Well-supported viral migration rates among countries estimated using different runs. The Bayesian Factor (BF) featuring each connection has been color-coded. Figure S3: Time-scaled maximum clade credibility trees obtained based on different datasets. The tree branches have been color-coded according to the most likely estimated country while the country posterior probability is displayed as a circle whose size is proportional to the probability value. Table S1: Dataset reporting the African strains used in the present study and the relative metadata. The Accession numbers of the strains obtained in the present study are also reported. Table S2: Dataset reporting the sequences used in the present study and the relative metadata. Table S3. Pairwise genetic distance calculated for both worldwide and African strains.
