# Supplementary figures and images for: A Phylogeographic Analysis of Porcine Parvovirus 1 in Africa
Source: Viruses. 2023 Jan 11;15(1):207. doi: 10.3390/v15010207 (PMC9864576; doi:10.3390/v15010207)

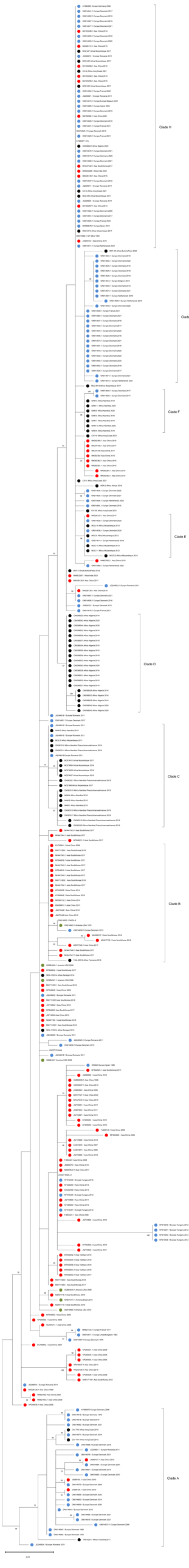

Supplement: Supplementary file 1 [file viruses-15-00207-s001.zip › Figure S1.pdf]

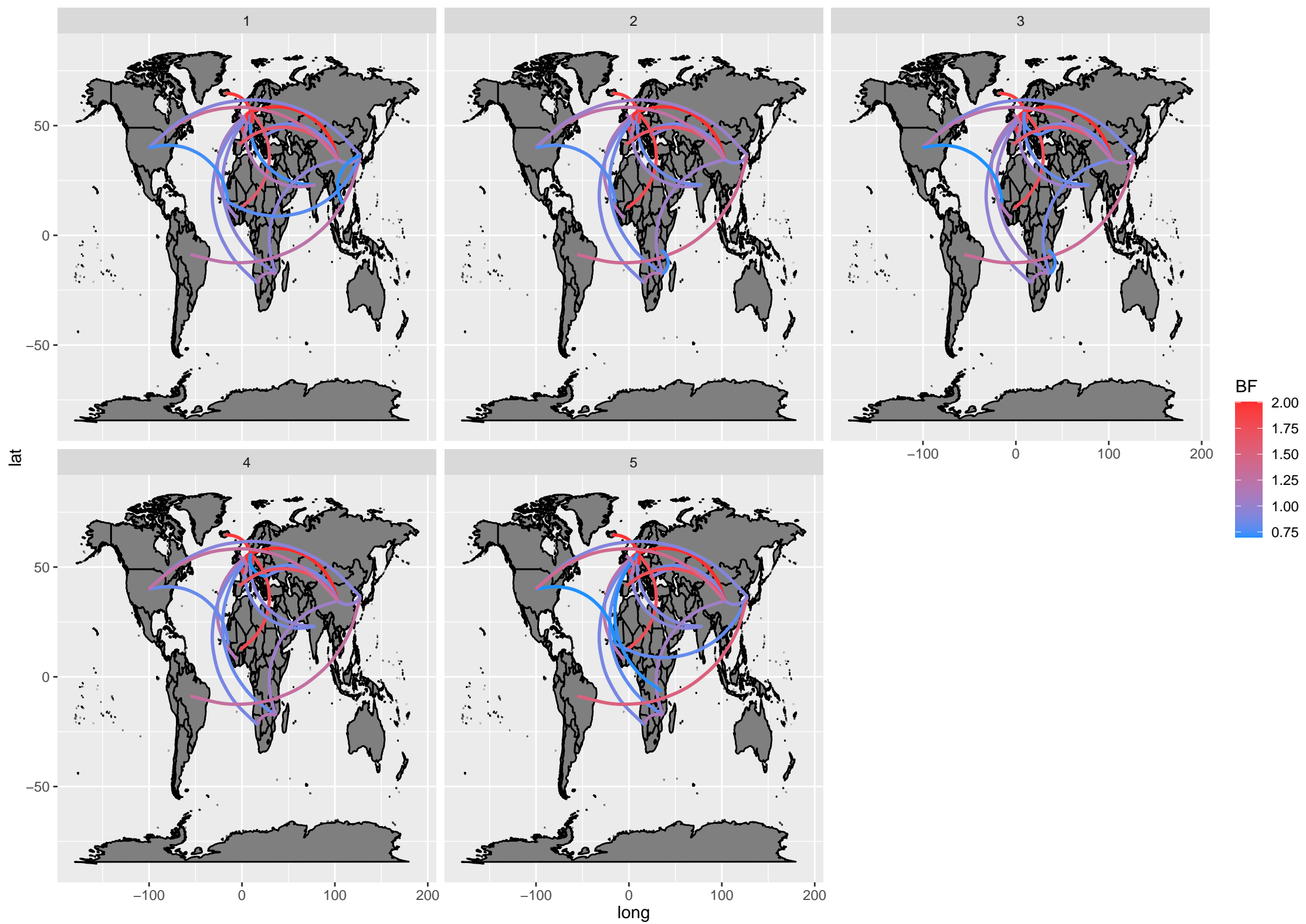

Supplement: Supplementary file 1 [file viruses-15-00207-s001.zip › Figure S2.pdf]

Dataset 1

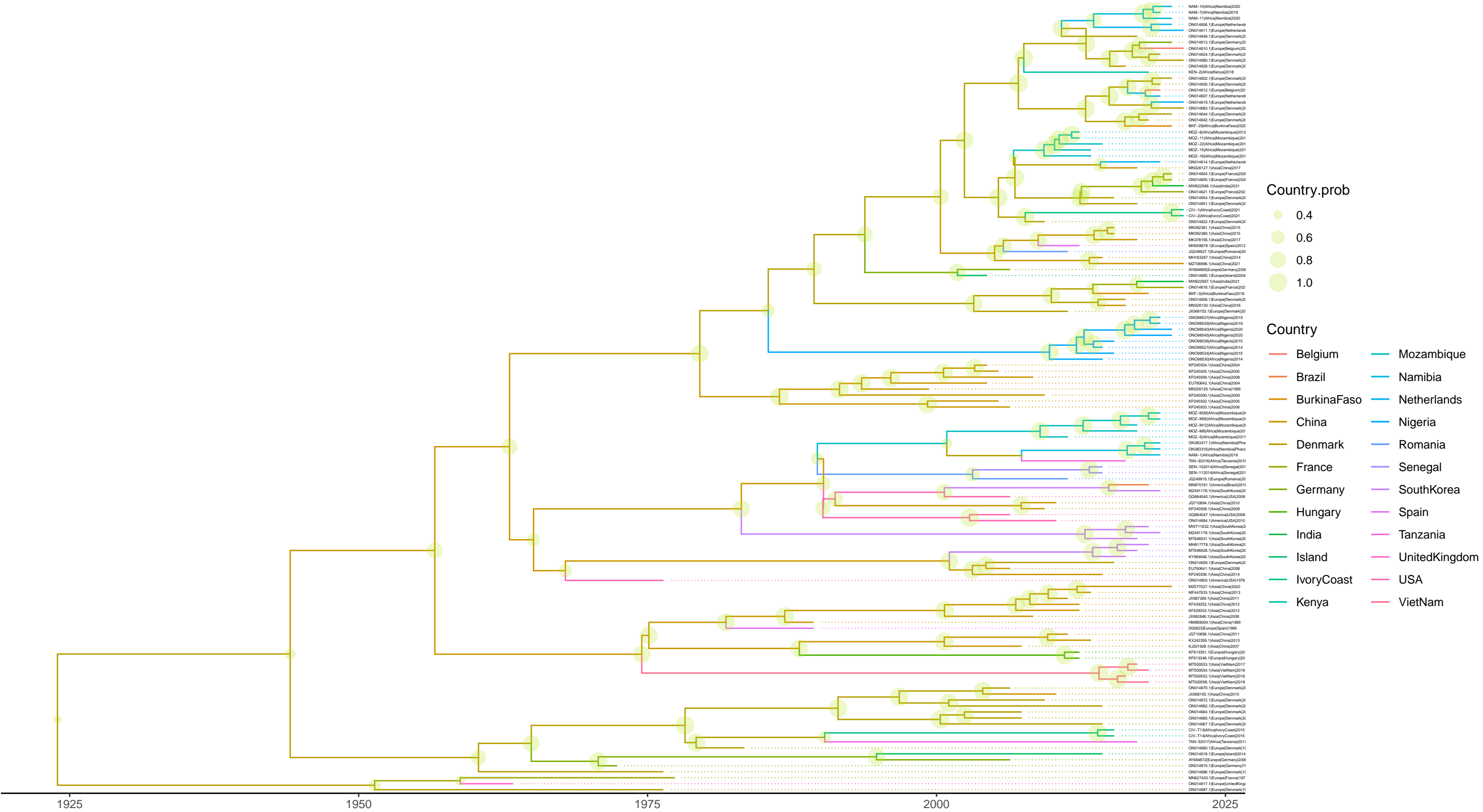

Dataset 2

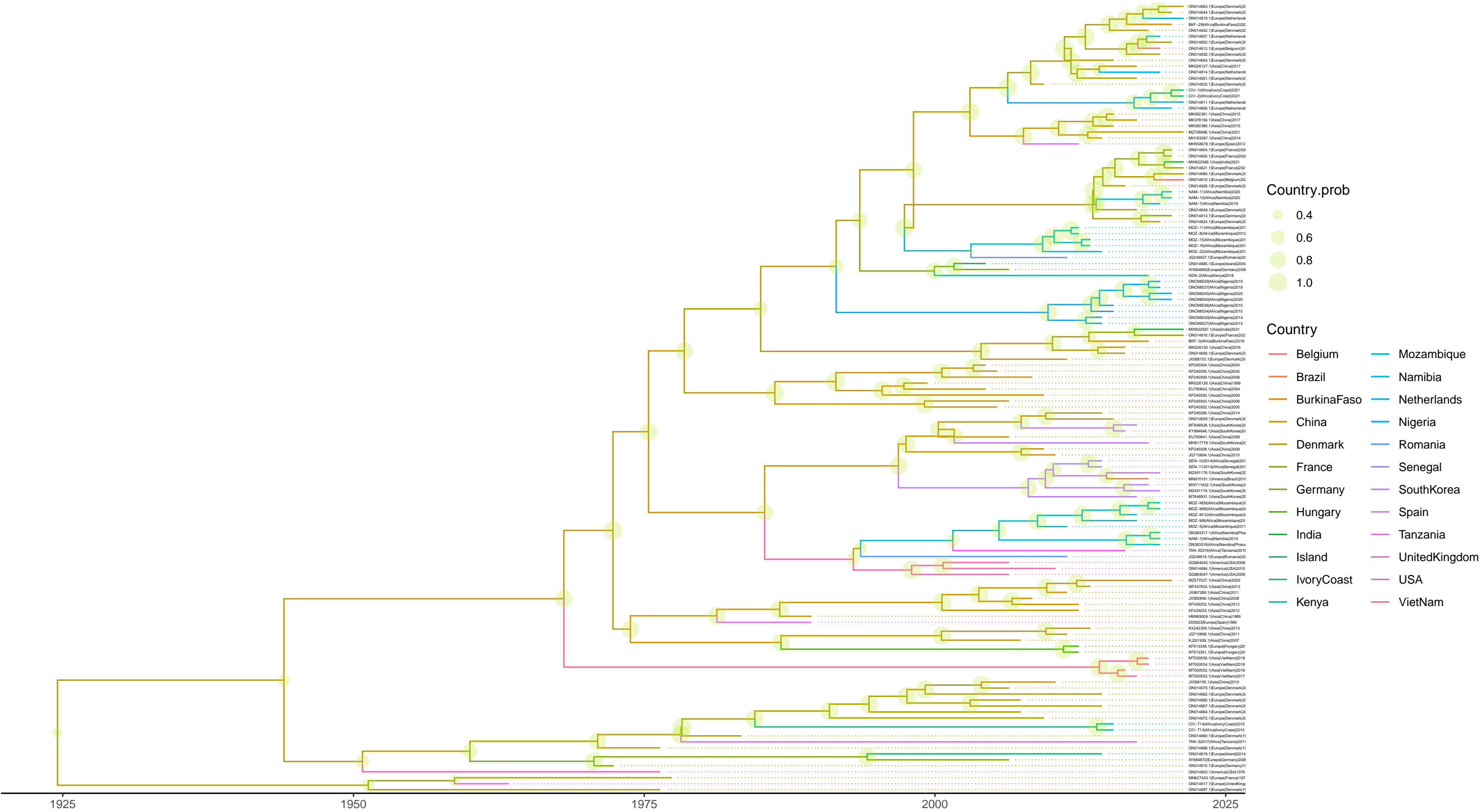

Dataset 3

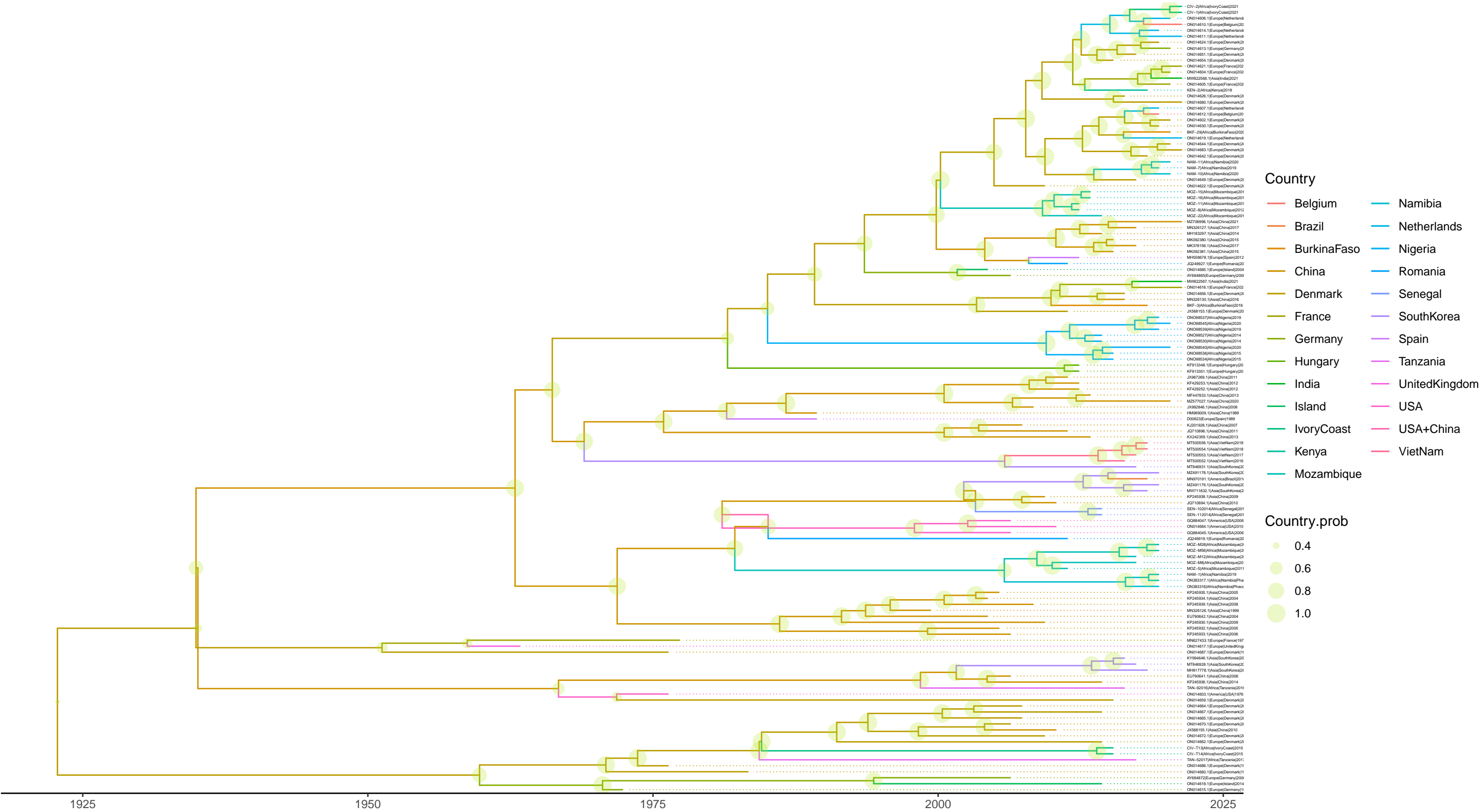

Dataset 4

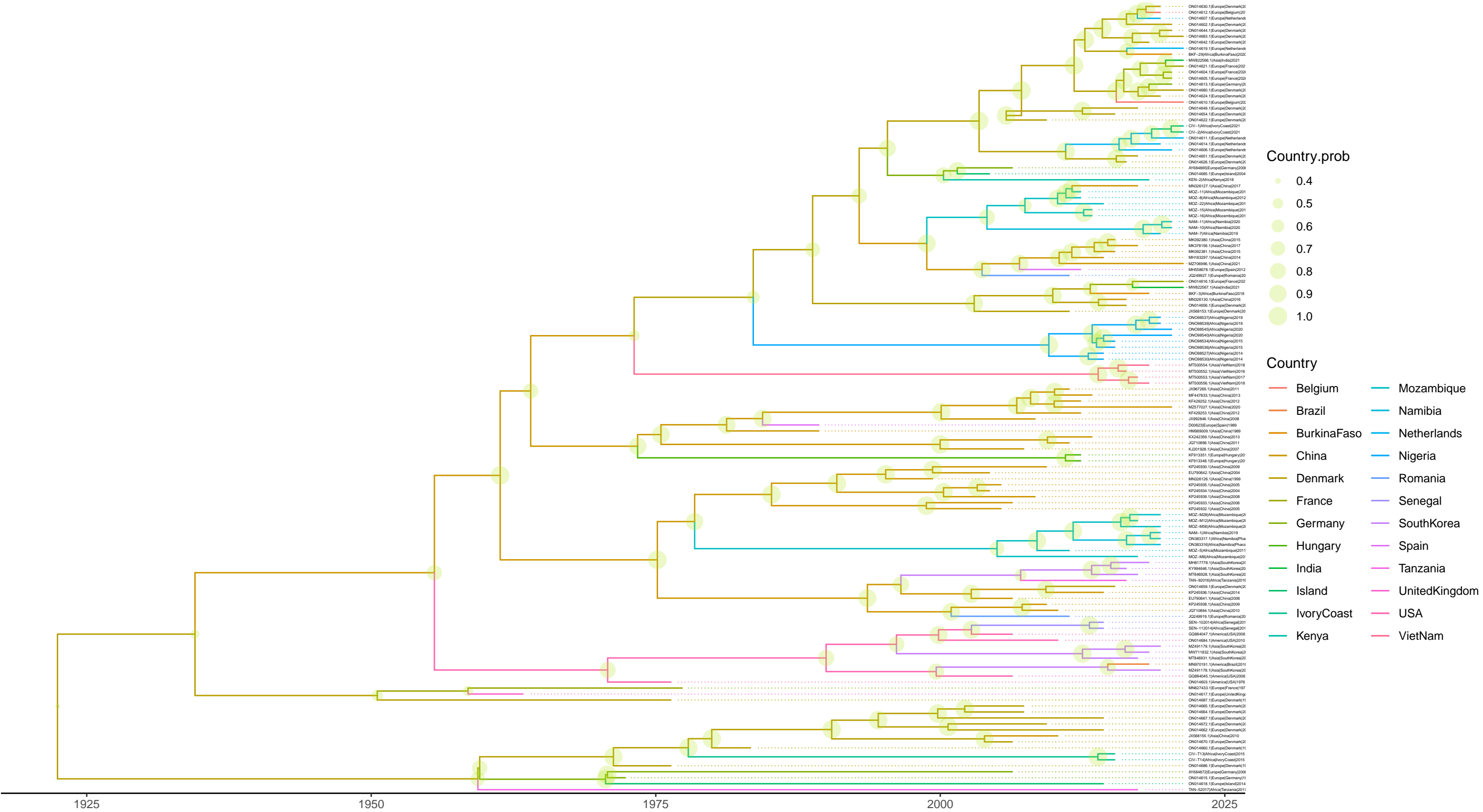

Dataset 5

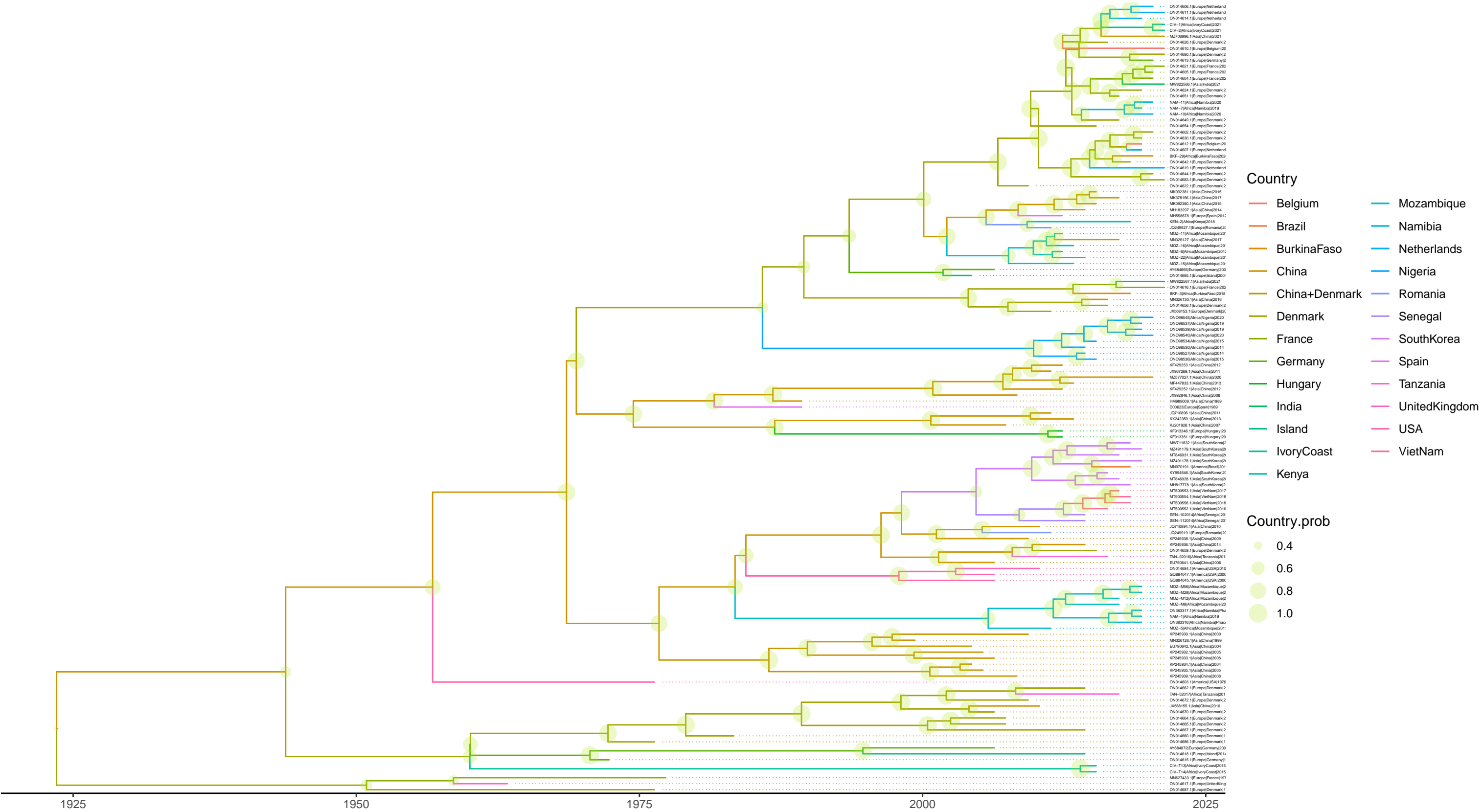

Supplement: Supplementary file 1 [file viruses-15-00207-s001.zip › Figure S3.pdf]
